# Should External Powered Orthoses be Used by Paraplegic Subjects or Not?

**DOI:** 10.5812/ircmj.3613

**Published:** 2013-06-05

**Authors:** Mohammad Taghi Karimi, Amir Esrafilian

**Affiliations:** 1 Department of Orthotics and Prosthetics, Rehabilitation Faculty of Isfahan University of Medical Sciences, Isfahan, IR Iran; 2 Department of Mechanics, Najafabad Branch, Islamic Azad University, Isfahan, IR Iran

**Keywords:** Orthotic Devices, Power, Spinal Cord Injuries

## Dear Editor,

Spinal cord injury (SCI) is a damage to spinal cord that results in loss of function, mobility and sensation below the level at which the spinal cord has been injured. The incidence of this disorder varies between 12.7 and 59 new cases per million each year (1). These subjects used various orthoses to improve their abilities to stand and walk, such as mechanical orthoses, functional electrical stimulation (FES), external powered orthoses (EPO) and hybrid system, which is a combination of FES and mechanical orthosis (2). However patients experience some problems such as high energy consumption during walking, high loads applied on upper limb and reduced walking speed (3). The external powered orthoses employ various kinds of power sources which include pneumatic, hydraulic and electrical power. Some orthoses such as pneumatic active gait orthosis (PAGO), powered gait orthosis (PGO), weight bearing control orthosis (WBCO), two degree of freedom orthosis (TDFO), driven gait orthosis (DGO), hybrid assistive limb (HAL) and lowered extremity powered exoskeleton (LEPE) have been designed to improve the performance of the subjects (4-10). However, the main questions posted here is how much the performance of paraplegic subjects is improved while walking with EPO in contrast to mechanical systems. Furthermore, it is not clear that how much is the willingness of subjects to use these orthoses (3).

**Figure 1.**
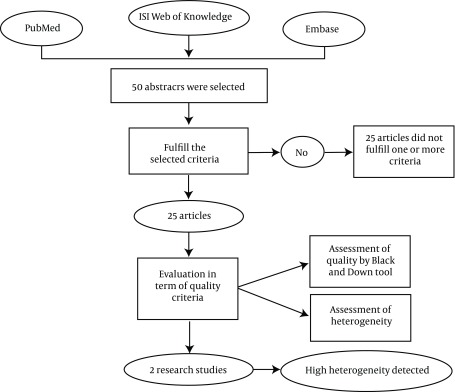
The Stages Which Were Selected in This Research Study

An electronic search was done via Pubmed, Embase and ISI web of knowledge data from 1960 to 2010. Some key words such as external power orthosis, spinal cord injury and rehabilitation were used. The abstracts and titles of each individual study were assessed by two reviewers based on whether the abstracts addressed the research question of interest, Figure 1. The quality of the studies was assessed by use of Black and Down tool. 

A total number of 25 relevant papers have been found based on aforementioned key words. After considering the relevant criteria only 5 papers were selected for final analysis. Most of the research focused on introducing the orthoses without undertaking any clinical studies. There were only 5 papers covered the data of gait analysis while walking with orthosis. The quality of studies based on Black and Down test was 4, 4, 16, 15, 6, 1, 1 and 1 for PAGO, PGO, WBCO, TDFO, DGO, HAL, Berkley and LEPE orthoses, respectively. Although various types of external powered orthoses have been designed for paraplegic subjects to improve their performances, there is not too much research to evaluate their performance. Moreover, the quality of available research is not acceptable. Two of the important parameters regarding the use of orthosis for paraplegic subjects are ease of donning and doffing of orthosis and also the cosmesis of orthosis (4). There are only two studies on WBC orthosis which show no difference between the performance of this orthosis and other available mechanical devices (6, 10).

Some devices such as HLO, hybrid assistive device and other lower extremity exoskeleton used mostly for military purposes. They have been designed to allow solders to carry injured solders and to bring food and first supplier to the area where vehicle cannot enter. Furthermore, they have noticeable problems including inadequate power supply, recharging batteries which require especial facilities and cost and size of the system. As there is no research evaluating the performance of orthosis with external powered supply on paraplegic subjects, it is recommended that the performance of these orthoses be evaluated by motion, stability and energy consumption analysis. Furthermore it is recommended that some parameters such as the willingness of subjects, ease of donning and doffing be considered in this regard.
